# The Photothermal Synergistic Mechanism of Rock Varnish Photoconductance Under Laser Irradiation

**DOI:** 10.3390/ma17235841

**Published:** 2024-11-28

**Authors:** Xinyang Miao, Tiantian An, Lijun Wang, Shujie Xiong, Huanxi Zhang, Jiahao Yi, Bingbing Zhao, Kun Zhao

**Affiliations:** Key Laboratory of Oil and Gas Terahertz Spectroscopy and Photoelectric Detection, Petroleum and Chemical Industry Federation, China University of Petroleum, Beijing 102249, Chinazhk@cup.edu.cn (K.Z.)

**Keywords:** rock varnish, photoconductance, laser, photothermal interactions

## Abstract

Rock varnishes, complex structures formed by long-term deposition on rocks, exhibit unique light absorption characteristics and are widely distributed across arid environments on Earth’s surface. The varnishes possess the ability to absorb and convert photons from solar radiation into electrons, which represents a newly discovered fundamental energy form in nature, with further elucidation required regarding the underlying mechanism of how semiconductor minerals respond to light radiation. The regulations governing the photoconductive responses of samples from the Alashan region in Gobi, China, and the mechanisms exhibited by rock rock varnishes under various bias voltages and irradiation wavelengths (532 nm, 808 nm, and 1064 nm) were studied. The photoconductivity response is positively correlated with the applied external bias, and the response caused by shorter wavelengths is larger. The synergistic effect was quantitatively assessed by monitoring and fitting the correlation between photoconductivity, temperature, and time during laser irradiation. As an effective method to study the fundamental physical properties of semiconductor minerals, the photoconductivity testing will help to establish a fundamental framework for investigating the intrinsic physical characteristics of natural rock varnishes.

## 1. Introduction

Earth’s surface has been continuously exposed to solar radiation for billions of years, facilitating photosynthesis and related biological processes in organic organisms [1]. Solar irradiation plays a crucial role in shaping Earth’s climate and chemical environment through both organic and inorganic mechanisms [2]. Rock varnish, also known as desert varnish, is a thin and dark coating observed on exposed surfaces of rocks in arid environments. It primarily consists of clay minerals, along with manganese and iron oxides [3,4]. Prolonged exposure to sunlight typically leads to the development of a lustrous black, red, or brown patina on these rock surfaces. This phenomenon is mainly observed on highly weathered rocks and remains unaffected by variations in lithology. The formation process of the varnish is complex and involves microbial activity, chemical deposition, and photochemical reactions. Notably, certain rock varnishes have been found to contain abundant natural semiconductor materials such as goethite, hydronatromanganite, and hematite; this abundance facilitates the occurrence of photoelectric conversion phenomena upon exposure to solar radiation [5,6,7,8]. The semiconductor materials incorporated into the varnish mimic the characteristics observed in biological photosynthesis, thereby facilitating efficient conversion of solar energy into chemical energy while simultaneously enabling oxygen production and carbon fixation [9,10]. Significant progress has been made in understanding the composition, morphology, and paleo climatological importance of rock varnishes in different regions, as well as their interactions with microbial communities. However, challenges persist in investigating the correlation between the photoelectric properties of rock varnishes and their elemental composition, as well as understanding the underlying response mechanism. Existing studies have primarily focused on validating the photoelectric properties of rock varnishes themselves without drawing definitive conclusions regarding the response mechanism or factors influencing response intensity [11,12,13].

In recent years, the investigation of laser–media interaction has emerged as a focal point in research. Utilizing advanced techniques such as laser-induced breakdown spectroscopy and time-of-flight testing, researchers have delved into the intricate mechanisms underlying plasma formation induced by lasers, their influence on electron density, and their interactions with matter [14,15]. Light excitation can effectively modulate the carrier concentration of materials, obviating the need for intricate adjustments in chemical composition or crystal structure. Upon laser irradiation, a cascade of physical phenomena is initiated, encompassing reflection, absorption, energy conversion, thermal effects such as heating and melting, and even gasification. The presence of an internal or external electric field in the material leads to the detection of laser-induced voltage (LIV) in the external circuit, attributed to the phenomena of photoelectric effect, photothermal effect, photoacoustic effect, and synergistic polarization charge [16]. The analytical technique enables precise regulation and characterization of a material’s physical properties based on the generation and transport characteristics of photogenerated carriers. Due to its non-destructive nature and high precision as a non-contact measurement method, LIV has extensive applications in various fields, including petrophysical, mineral property detection, as well as the sustainable exploration of unconventional resources [17]. By examining the photoelectric characteristics and response mechanism of rock varnishes collected from the Alashan region in China, this study compares their morphology and composition on rock surfaces and inside rocks based on elemental compositions. Additionally, we investigate the regulations governing photoconductive responses and mechanisms exhibited by rock rock varnishes under different bias voltages and irradiation wavelengths. Our objective is to establish a fundamental framework for investigating the intrinsic physical characteristics of natural rock varnishes, thereby enhancing our comprehension of their pivotal role in geological evolution.

## 2. Materials and Methods

The experimental specimens utilized in this study were collected from the Alashan region of the Gobi Desert, China. Over time, these rocks develop a distinct “patent skin” due to prolonged exposure to sunlight, erosion caused by sand and salt, and the impact of storms [3,10]. This process results in a smooth and lustrous appearance on the sun-facing side where they come into contact with the ground. In contrast, the interior of unexposed rock lacks such prolonged direct sunlight exposure and appears rough and lackluster. X-ray diffraction (XRD) is used to determine the mineral composition in rocks. To gain deeper insights into the morphology and composition of rock varnish on these rocks, scanning electron microscopy combined with energy-dispersive X-ray spectroscopy (EDS) was employed for sample analysis. The samples used for the tests mentioned above were regular-shaped cut blocks, and the tests were conducted at room temperature.

**Figure 1:**
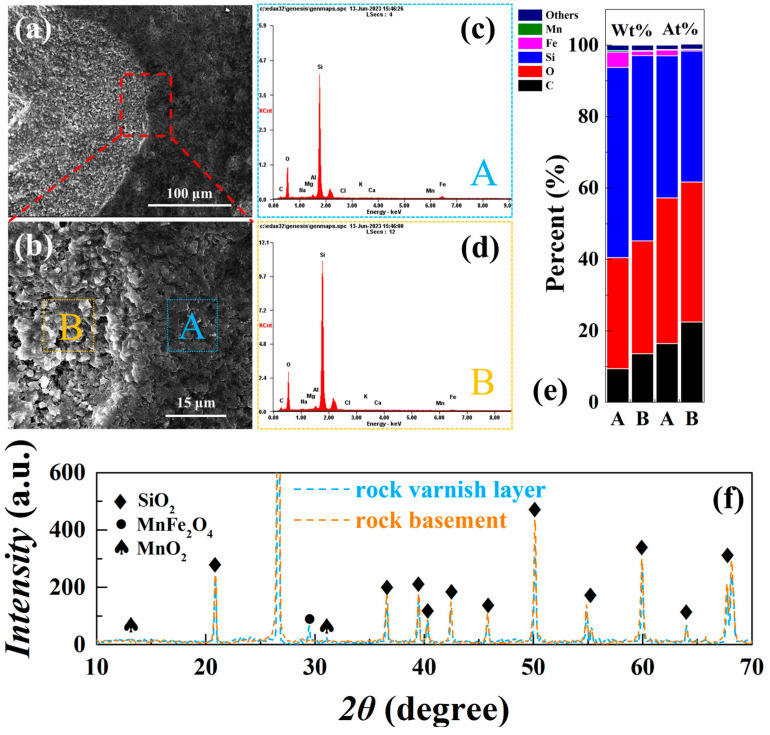
Basic characterization of rock varnish sample cutting. (**a**) SEM topography of the interface between the rock varnish layer and the rock basement; (**b**) locally enlarged topography; EDS spectra of the rock varnish layer (**c**) region A and (**d**) region B. (**e**) Distribution of elements in region A and inner region B, including mass percentage and atomic percentage. (**f**) XRD results for both the rock varnish layer and rock basement.

The interfacial morphology of the rock varnish/interior is shown in Figure 1a, with the rock substrate on the left and the rock varnish covered by surface deposition on the right. The figure clearly demonstrates distinct boundaries between these two areas. Compared to the dark and smooth varnish layer, the rock basement exhibits a more rugged surface structure with unevenly distributed micron-scale pores. Upon zooming in on a local section of Figure 1b, various depressions and protrusions can be observed on its surface, varying in size, depth, and form, indicating the relatively dense nature of the rock varnish deposited on top of the rock. The SEM image reveals a denser texture of the rock varnish compared to the rock matrix, characterized by a smoother surface and darker color. Its thickness measures approximately 2 μm, consistent with findings from previous studies [18]. The energy spectra analysis for both regions (Figure 1c,d) shows that silicon, oxygen, and carbon are the major components in both rocks’ rock varnish and inner substrate. Aluminum, on the other hand, constitutes a relatively small proportion (~1%), with similar contents across both regions. The main difference lies in the content of iron and manganese, which are semiconductor minerals found in the rock varnish layer. We conducted X-ray diffraction (XRD) tests on the sample coating and internal matrix layer separately. The results revealed quartz as the main mineral inside the sample, with a small amount of iron manganese oxide present in the surface varnish layer. The source of the manganese and iron remains uncertain. It is widely accepted that surface weathering releases Mn^2+^ ions from dust, while subsequent physicochemical, biochemical, and photochemical processes collectively facilitate their oxidation and enrichment [19,20,21]. These iron and manganese minerals are considered important sources of photogenerated charge carriers under illumination [2,3,4].

**Figure 2:**
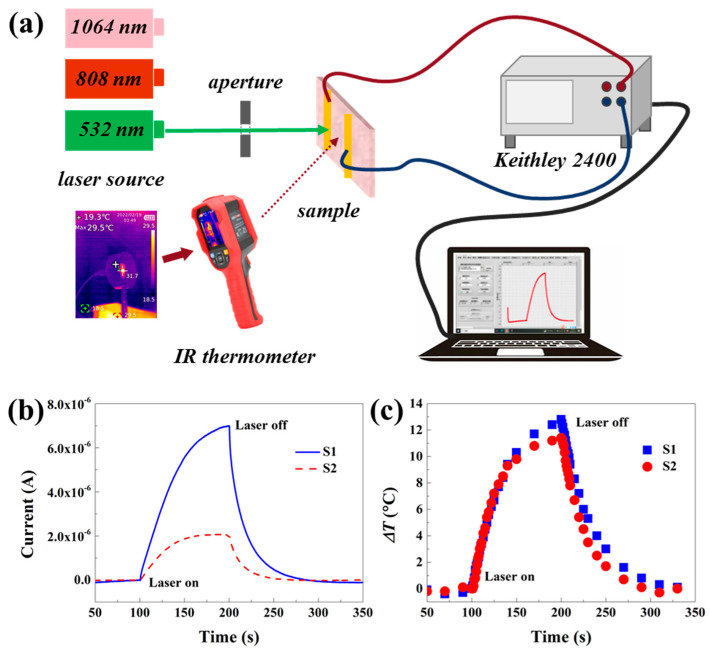
(**a**) Schematic diagram of laser irradiation test device for rock varnish. The relationship of (**b**) current and (**c**) temperature of S1 and S2 samples with time under laser irradiation; 532 nm laser turned on at 100 s and turned off at 200 s.

Two cuboidal samples, measuring 7 × 4 × 2 mm^3^, were extracted from the same rock formation. The upper surface of sample S1 displayed a rock varnish, while both the surface and interior of sample S2 were entirely free of any rock varnish. Two silver electrodes were fabricated and interconnected using a copper wire, each with a length of 4 mm and a separation distance of 3 mm between them. Three solid-state lasers emitting continuous light at wavelengths of 532 nm, 808 nm, and 1064 nm were selected as the laser sources. Figure 2a illustrates the experimental setup featuring two apertures (3.8 mm in diameter) positioned between the light source and the sample to ensure precise optical path alignment. The copper wire was connected to a Keithley 2400 digital source meter via BNC connection cables for real-time recording of optical response signals generated by the samples during laser irradiation tests. Upon exposure to laser irradiation, materials undergo processes such as absorption, reflection, and transmission on their surfaces and within their interiors that yield electrical responses including photovoltage, photocurrents, and photoconductivity. Among them, photoconductance refers to the phenomenon of enhanced conductivity under light [17,18,19,20]. Figure 2b illustrates the photoconductance response of a sample with a rock varnish (S1) and a rock base sample (S2) under laser irradiation at 532 nm. As the laser irradiation progresses, the sample’s photoconductive response rapidly increases, resulting in a significant rise in current and eventually reaching a plateau after a certain period. Upon laser deactivation, there is a sudden decline in response. A comparison of the photoconductive responses of both samples reveals that the current increase on the rock varnish surface is more pronounced than within the rock substrate. This difference can be attributed to variations in composition and internal structure between these two materials. The varnish contains a higher abundance of semiconducting minerals compared to the rock substrate. Additionally, aluminum acts as a P-type dopant to enhance electrical conductivity and photoelectric response, while transition metal elements like Mn and Fe absorb incident light energy, promoting electron excitation and modifying the energy level distribution for improved photoconductive response.

To effectively monitor this change, direct an infrared imager lens towards the central region where light irradiation occurs and record real-time temperature variations under laser irradiation. Figure 2c demonstrates simultaneous monitoring of temperature changes alongside photoconductivity phenomena. It can be observed that both samples exhibit similar trends in terms of temperature variation within a range of 11 °C to 15 °C. The photon energy from a laser is absorbed and converted into thermal energy upon irradiating the surface of a semiconductor material, resulting in an overall increase in temperature through heat conduction, diffusion, and convection mechanisms. Simultaneously, the alteration in temperature influences the semiconductor properties of the material.

## 3. Results and Discussion

**Figure 3:**
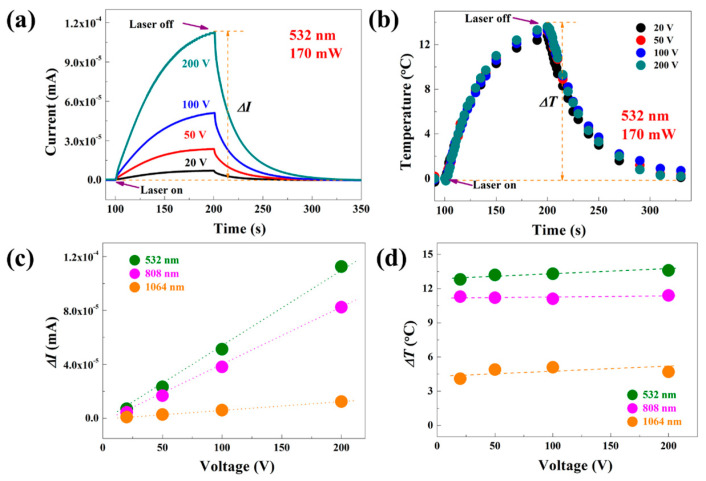
(**a**) Photoconductivity waveform and (**b**) temperature–time curve of rock varnish samples irradiated by 532 nm laser at different bias pressures. The relationship between bias and (**c**) Δ*I* and (**d**) Δ*T* under different laser irradiation.

The rock and rock varnish samples, acting as semiconductor materials deposited by sunlight over an extended period, exhibit photon energy absorption under laser irradiation. The process stimulates electron transition and generates additional charge carriers that move directionally in response to an electric field, resulting in a photoconductive response. Initially, the influence of bias voltage on both photoconductivity response and temperature was investigated. Different bias voltages (+20 V, +50 V, +100 V, and +200 V) were applied to the laser-irradiated rock rock varnish samples while monitoring the sample surface temperature. The amplitude of the photoconductivity response increases proportionally with an increase in bias voltage, as illustrated in Figure 3 for the case of 532 nm laser irradiation. Higher bias voltages result in an accelerated rate of photoconductance rise. The variation in surface temperature (Δ*T*) remains insignificant across different biases. The photoconductivity of the material is influenced by the applied external bias voltage, which modifies the internal electric field intensity, charge carrier distribution, and migration rate. The change in current (Δ*I*) exhibits a positive correlation with the bias voltage. Due to the high resistance of the sample, the Joule thermal effect induced by the current is not prominent, resulting in insignificant influence on the surface temperature of the sample caused by variations in bias voltage. The influence of irradiation wavelength on photoconductivity response was further analyzed while maintaining a constant laser power of 170 mW for each type, under stable conditions with fixed sample position and spot location. The relationship between bias voltage and Δ*I*, as well as Δ*T* for different laser wavelengths, is demonstrated in Figure 3c under three distinct laser irradiations. Under identical laser power, shorter wavelengths correspond to higher photon energy, leading to larger amplitudes of the photoconductive response when illuminating samples. Additionally, there exist disparities in the amplitude of Δ*T* among samples exposed to three distinct laser wavelengths: 532 nm demonstrates the highest magnitude of temperature alteration, followed by the 808 nm wavelength, while minimal temperature variation is observed at 1064 nm.

**Figure 4:**
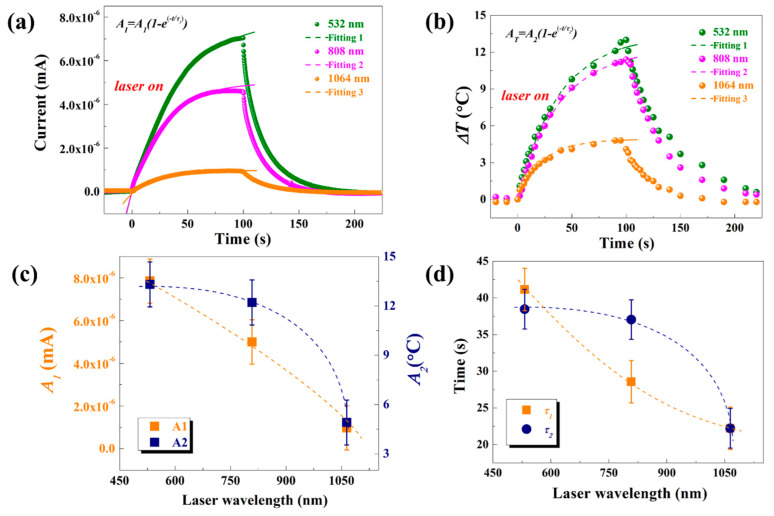
Exponential fitting of (**a**) photoconductive response rise process and (**b**) temperature change rise process of rock varnish samples irradiated by laser at different wavelengths. The fitting parameters under different wavelength laser irradiation include (**c**) coefficients *A*_1_, *A*_2_ and (**d**) time constants *τ*_1_ and *τ*_2_.

The waveforms of the signals under different laser irradiation were further analyzed, with a fixed bias voltage set at 20 V. To investigate the mechanism behind the influence of various laser wavelengths on the photoconductivity response of rock varnish samples, the measured waveforms of Δ*I* and Δ*T* were characterized well by the following exponential functions, as indicated by the dashed line in Figure 4.

(1) $$\Delta I=A_{1}(1-\exp(-T/\tau_{1}))$$

(2) $$\Delta T=A_{2}(1-\exp(-T/\tau_{2}))$$

The fitted curve coincided with the original response curve, both exhibiting an initial rapid rise followed by a slower increase until reaching equilibrium state. The fitting coefficients are presented in Figure 4c,d. *A*_1_ and *A*_2_ represent stable values reached by current and temperature during the rising stage under three laser wavelengths respectively, while *τ*_1_ and *τ*_2_ denote time constants indicating the duration required for the signal to reach a certain stable value. It can be observed that as the laser wavelength increases, both the photoconductance response under laser irradiation and the steady-state temperature change decrease. The time constant represents the duration required for the signal to reach equilibrium from its initial stage. At a laser wavelength of 1064 nm, it is evident that both photoconductance and temperature change reach equilibrium simultaneously. However, when exposed to a 532 nm laser, the temperature change reaches equilibrium later than the photoconductance response, indicating a delay in reaching equilibrium for the temperature change compared to the photoconductance response. Conversely, the equilibrium of the photoconductance response is achieved earlier than that of the temperature change under 808 nm laser irradiation. These phenomena are primarily influenced by the energy absorption efficiency, carrier production efficiency, and carrier recombination processes in rock varnish materials. Firstly, lasers with different wavelengths exhibit varying energy absorption efficiencies in rock varnish materials. Secondly, lasers of different wavelengths have diverse effects on charge carrier production efficiency; higher photon energies facilitate easier generation of electron-hole pairs, resulting in greater carrier concentrations. Finally, both photoconductive responses and the amplitude of temperature changes in rock varnish materials are affected by charge carrier recombination processes.

**Figure 5:**
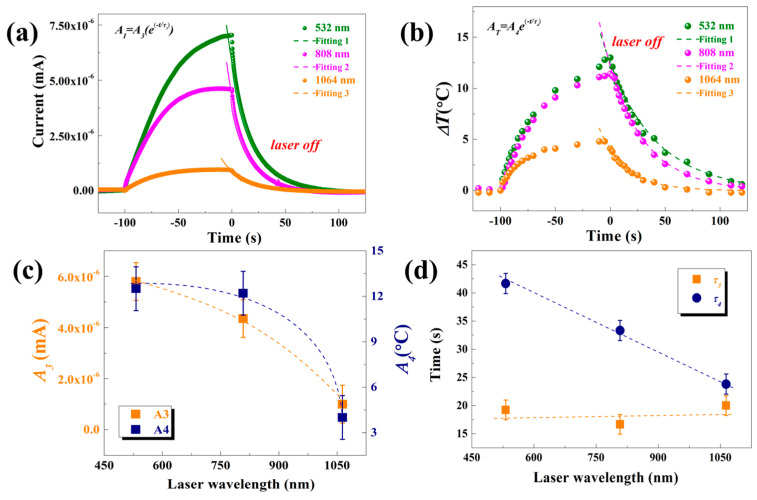
Exponential fitting of (**a**) photoconductive response decline process and (**b**) temperature drop process of the sample after laser irradiation is stopped. The fitting parameters under different wavelength laser irradiation include (**c**) coefficients *A*_3_, *A*_4_ and (**d**) time constants *τ*_3_ and *τ*_4_.

After the cessation of laser irradiation, both the photoconductance response and temperature change on the sample surface enter a declining stage. The fitting of this declining stage is illustrated in Figure 5 by the following exponential function, demonstrating that the black fitting curves perfectly align with the original response curve. In order to establish a consistent reference point, we have shifted the initiation of the descent process to zero.

(3) $$\Delta I=A_{3}\exp(-T/\tau_{3})$$

(4) $$\Delta T=A_{4}\exp(-T/\tau_{4})$$

Initially, there is a rapid decrease, followed by a gradual stabilization until reaching an equilibrium state. Similarly, during this declining stage under laser irradiation at three different wavelengths, stable values (*A*_3_ and *A*_4_) for current and temperature, respectively, are reached along with corresponding time constants (*τ*_3_ and *τ*_4_). These results are presented in Figure 4. It can be observed from the figure that both *A*_3_ and *A*_4_ decrease as the laser wavelength increases. Comparing these values to their respective time constants reveals that all three wavelengths exhibit similar time constants (*τ*), which are smaller than *τ* overall, indicating that the current curve synchronizes during the decline process, while temperature changes take longer to reach equilibrium state restoration. Furthermore, comparing these values to Δ*T* demonstrates that after ceasing irradiation, temperatures begin to decline from their highest states associated with each laser wavelength. Notably, due to its smallest amplitude of temperature change caused by 1064 nm laser irradiation, it restores its initial state fastest.

**Figure 6:**
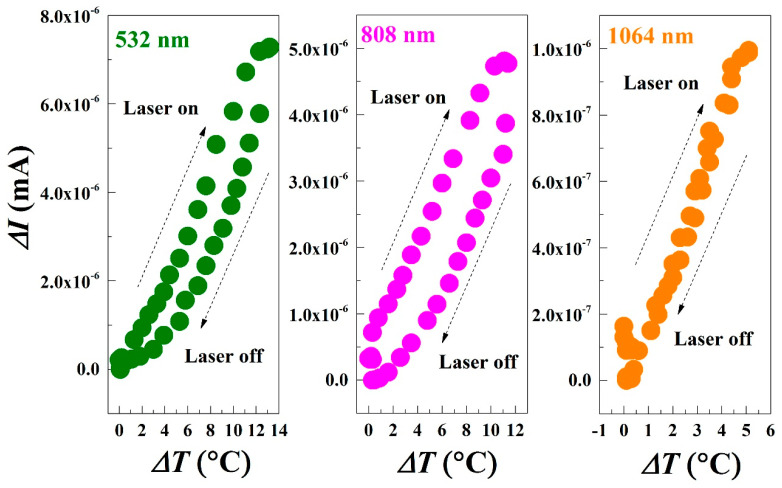
The relationship between Δ*I* and Δ*T* at different laser wavelengths.

Based on the aforementioned results, it is evident that the photoconductance time curve and temperature–time curve exhibit variations in amplitude and the rising and falling speed under different laser irradiation conditions. To further elucidate their relationship, simultaneous extraction of corresponding Δ*I* and Δ*T* was performed for three distinct laser wavelengths. The analysis of their interrelationship yielded the outcomes depicted in Figure 6.

As depicted in the figure, a positive correlation between current and temperature is observed. Specifically, for the 532 nm laser wavelength compared to the 808 nm laser wavelength, data points exhibit a clockwise ring pattern with non-coincident curves for temperature rise and fall. This suggests that under identical temperatures, illuminated conditions consistently yield higher currents than non-illuminated conditions. Upon absorption of photons surpassing the bandgap threshold energy, electrons transition from lower-energy valence bands to higher-energy conduction bands while leaving behind holes in valence bands. Activation of the laser leads to a rapid increase in current; conversely, deactivation results in swift current decrease followed by gradual decline as the temperature decreases. These findings align with fitting parameter laws governing signal waveforms and further substantiate that both photogenerated carriers and photothermal processes influence rock varnish conductivity. In contrast, under 1064 nm laser irradiation, coincident curves for temperature rise and fall indicate that sample photoconductance solely relies on temperature.

Rock varnishes possess significant research value across various fields. Sunlight, which is nearly omnipresent, serves as both the ultimate energy source for the biosphere and the driving force behind surface and lithospheric movements. Advancements in science and technology have enabled humans to harness light energy through diverse devices. Photosynthesis allows plants to directly convert light energy into bioenergy, which subsequently flows through ecosystems and is ultimately utilized by humans. Recent studies have shown that natural semiconductor minerals possess stable and sensitive photoelectrical conversion properties under radiation, resembling the oxygen production and carbon fixation systems of biological photosynthesis. While most research has focused on the photoelectric effect and photosynthesis-like effect of rock varnish, there is a lack of reported findings regarding the presence of photothermal effects in this process [2,3,4,5,6,7,8]. In this study, we focus on exploring the electrical properties of rock varnishes under laser irradiation using 532 nm, 808 nm, and 1064 nm lasers as light sources. By monitoring the relationship between photoconductivity, temperature, and time during laser irradiation, we observe and evaluate light–thermal synergies. Our objective is to establish a foundation for studying fundamental physical properties of natural rock varnishes while characterizing their roles in geology and biological evolution. Significantly, the interplay between the rock and the mineral varnish can yield diverse outcomes, which are influenced by factors such as the composition, structure, and morphology of the rock. Future investigations can delve into photoelectric properties such as absorption spectrum analysis, photoconductivity measurements, and carrier lifetime assessments of rock varnishes via spectral analysis techniques and electrochemical testing methods to explore their relationships with material composition and structure.

## 4. Conclusions

The photoconductivity mechanism of natural rock varnish was investigated in this study. It was observed that the electrical conductivity increased under laser radiation at wavelengths of 532 nm, 808 nm, and 1064 nm due to a synergistic contribution from the photothermal effect. This synergistic effect was quantitatively evaluated by monitoring and fitting the relationship between photoconductivity, temperature, and time during laser irradiation. At 532 nm and 808 nm, both mechanisms interacted synergistically to cause the photoconductive effect. At 1064 nm, changes in conductivity were solely influenced by a pure photothermal effect resulting from an increase in temperature. Photoconductivity testing serves as an effective approach to investigate the fundamental physical properties of semiconductor minerals, thereby facilitating their broader applications in the field of rock mineralogy.

## Data Availability

The original contributions presented in this study are included in the article. Further inquiries can be directed to the corresponding author.
